# Supplementary material for: Endogenous Signaling Molecule Activating (ESMA) CARs: A Novel CAR Design Showing a Favorable Risk to Potency Ratio for the Treatment of Triple Negative Breast Cancer
Source: Int J Mol Sci. 2024 Jan 3;25(1):615. doi: 10.3390/ijms25010615 (PMC10779313; doi:10.3390/ijms25010615)
Supplement: Supplementary file 1 [file ijms-25-00615-s001.zip › ijms-2775208-supplementary.pdf]

International Journal of
Molecular Sciences

MDPI

Supplementary material

Supplementary Method S1: Data analysis with the MACSPlex InspectoR web app

For the automated data analysis of measurements with the MACSPlex Cytokine 12 Kit, human, obtained with a MACSQuant flow cytometer (platform independent), the software MACXPlex InspectoR, Version 0.7.1 was used. It was developed with R version 4.1.3 (2022-03-10) and packages therein. The app was deployed on a local linux server (Platform: x86_64-pc-linux-gnu (64-bit) running under Ubuntu 20.04.4 LTS). The analysis method follows mainly the MACSQuant Analyzer Express Modes in MACSQuantify (software version 2.13.1) with a few amendments as detailed below.

In a typical workflow, first, after accessing the Web application , the MACSPlex Kit and the mqd files of the standards' measurements and of the sample need to be selected to start the analysis. The remaining analysis is then performed automatically. The results are displayed in the web app and include a figure of the singlet gate, an interactive scatterplot of the analyte bead populations, an interactive plot of the standard curves for all analytes, and a results table displaying the concentrations of the cytokines of the samples (**Supplementary Figure S1**). The results table can be explored interactively in the web ap-plication, or downloaded together with supplementary information (concentration of the samples, counts of beads per analyte cluster, median intensity values of the APC channels of the sample or the standard dilution series, and metadata).

The software to generate the results was programmed to resemble the MACSQuant Analyzer Express Mode for all functions except the analyte identification and assignments. First, a singlet gate is set on the FSC-A and SSC-A channels to select only single bead events, debris is additionally removed by exclusion of all intensities lower than 1 in the B1-A and B2-A channels. Next, clustering is applied to identify the bead populations for all analytes. The number of detected clusters is restricted to the number of analytes detectable by the selected kit. Once the populations are detected, they are assigned to the corresponding analytes. This is achieved by aligning the median intensities of each cluster as closely as possible to a grid of the theoretical ideal B1/B2 intensities of the kit. For each assigned analyte the correct neighbors are either confirmed, or the cluster centers are randomly shifted and the assignment procedure applied again. The assignment of the bead populations according to the analytes is performed in the same manner on both, the standards and the sample files.

The calculation of the standard curve was adapted from the MACSQuant Analyzer Express Mode by combining a local hermite fit with a linear interpolation. The concentrations of the dilutions are calculated with this standard curve function, and subsequently, the concentrations of the analytes measured in the samples are calculated accordingly.

The MACSPlex InspectoR application is deployed internally on a linux server.

Supplementary Figures

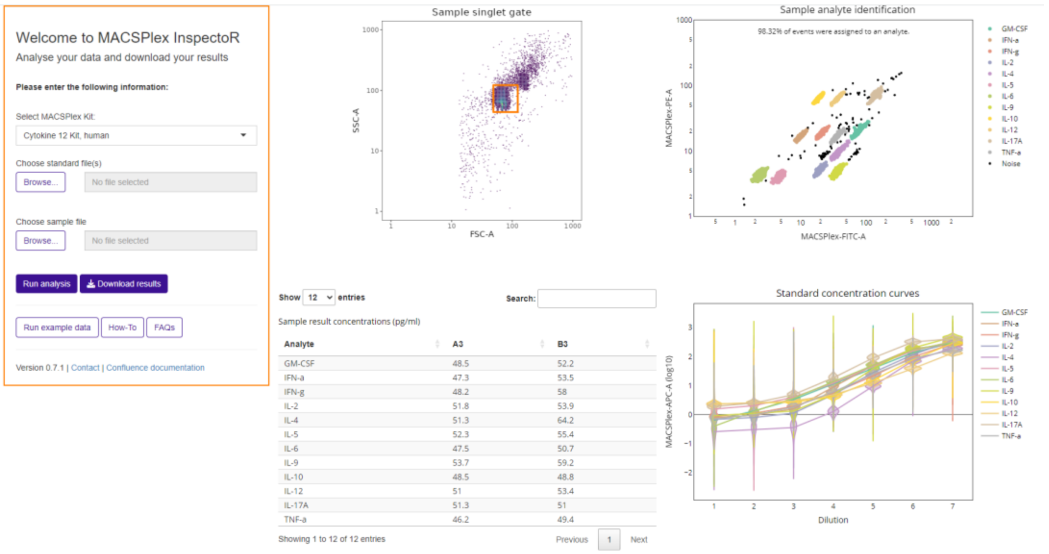

Supplementary Figure S1: MACSPlex InspectoR web application for the analysis of the MACSPlex Cytokine Kits. Exemplary screenshot of the MACSPlex InspectoR web application of the analysis of mqd files generated by measurements of samples with the MACSPlex Cytokine 12 Kit, human. On the left side is the selection panel to select the cytokine kit to be analyzed, the standards and sample files, and additional buttons to start the analysis, or download the results. The top middle plot displays the selection of the singlet gate, top right is the interactive plot to visualize which events were assigned to which analytes, the lower right interactive plot allows to explore the standard curve measurements, and the interactive table at the bottom displays the concentrations of the measured cytokines for each sample.

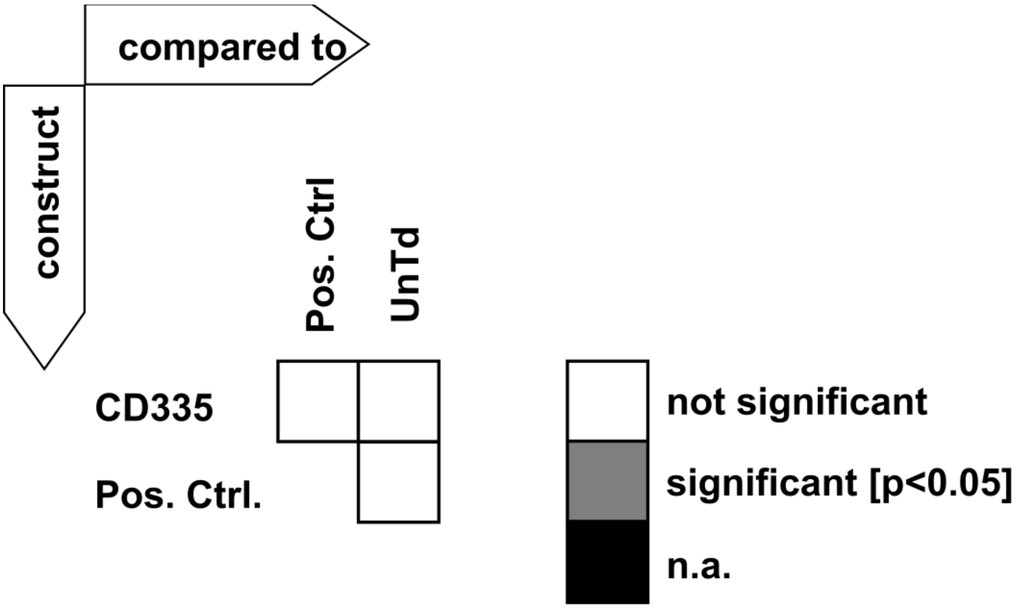

Supplementary Figure S2: Organization of the pairwise significance matrix (PSM) for group comparisons. Statistical significance assessed with one-way or two-way ANOVA (multiple comparisons), outcomes for each timepoint arranged in a PSM. $p \leq 0.05$ (grey), $p > 0.05$ (white), not assessable (n.a.) as $n < 3$ for at least one group (black).

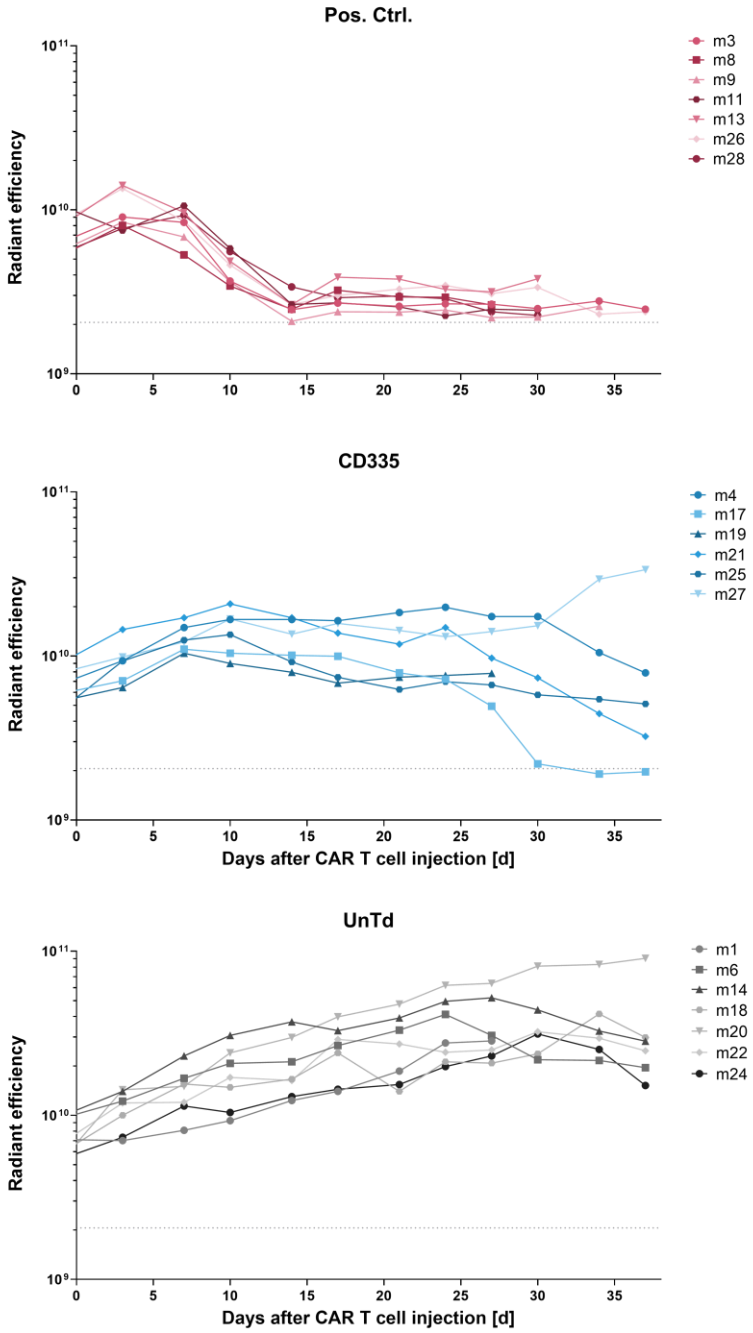

Supplementary Figure S3: Tumor burden per animal measured by fluorescence intensity over time. Tumor burden was monitored longitudinally by *in vivo* fluorescence imaging (FLI) of fluorescent reporter protein (TurboRFP) expressing MDA-MB-231 cells in each group. For detection of tumor burden variances within one group, individual values were compared for each group, respectively. Pos. Ctrl (n=7), CD335 (n=6), UnTd (n=7).

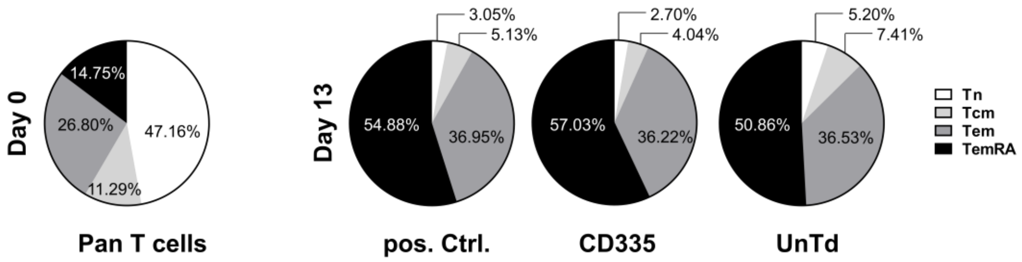

Supplementary Figure S4: Composition of different memory T cell subtypes before and after T cell expansion. Pan T cells were characterized by memory marker expression on day of isolation (day 0) by flow cytometry. Transduced CAR T cells and respective untransduced (UnTd) control T cells were stained for same markers after expansion on the day of *in vivo* injection (day 13). Percentages of naïve (Tn, CD45RA+CCR7+), central memory (Tcm, CD45RA-/CCR7+), effector memory (Tem, CD45RA-/CCR7-) and terminally differentiated effector memory T cells (TemRA, CD45RA+/CCR7-) are depicted.

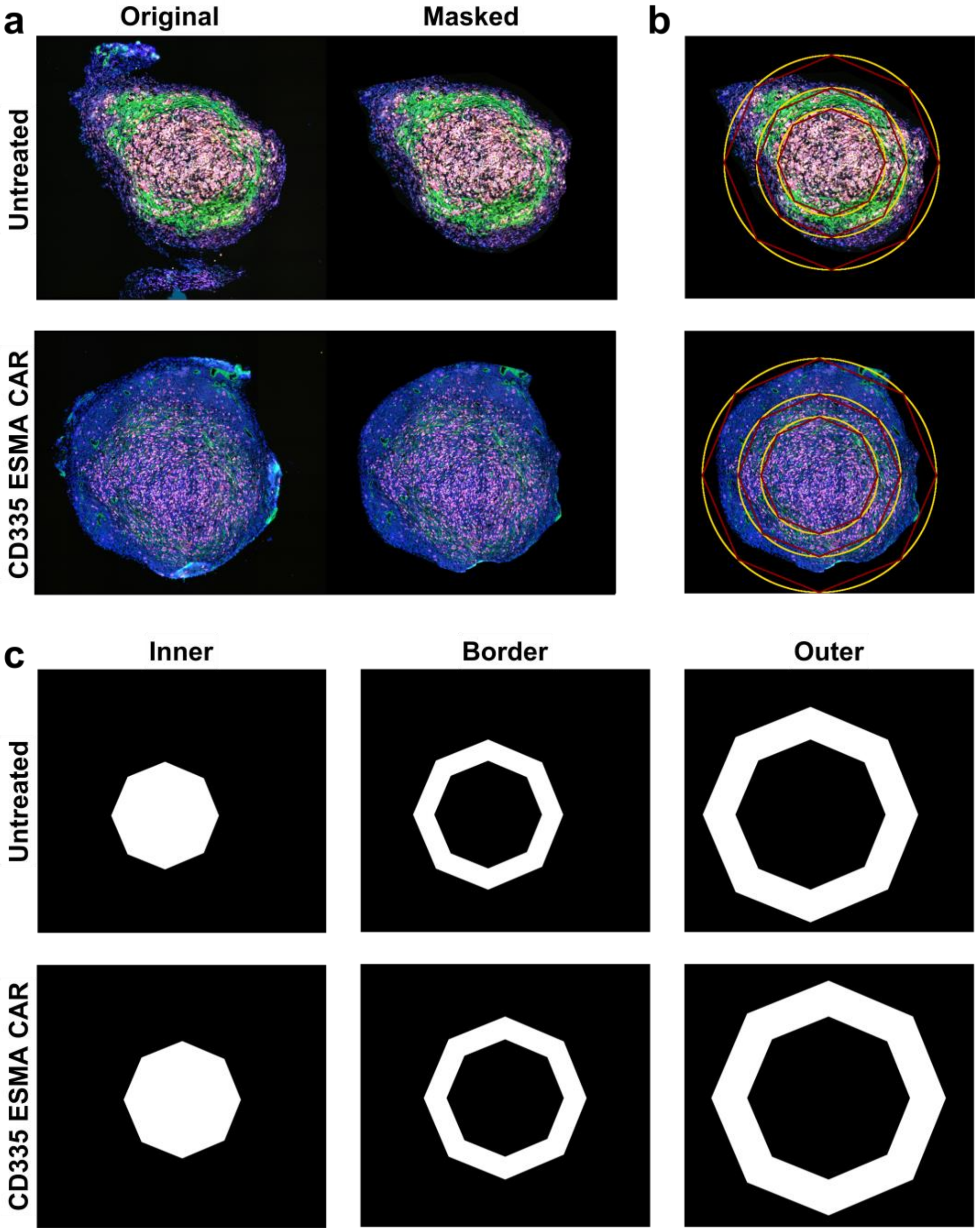

Supplementary Figure S5: Tumor segmentation into distinct regions to analyze intra-tumoral target and T cell distribution. Tumors imaged using the ultrahigh-content MACSima™ Imaging Platform for deep phenotyping of tumor cells and infiltrating T cells were partitioned into three regions to analyze intra-tumoral cell distribution. Tumor regions were defined for CD335 ESMA CAR treated tumor (n=1) and untransduced (UnTd) control (n=1) separately using the same procedure. (**a**) Mask-ing of tumor border parts with oversaturated intensities and imaging artifacts due to overlapping tissues was performed. (**b**) The tumor was partitioned into an inner tumor core region, an outer surrounding region and a border region in between. Partitioning was performed by creating a circle delimiting the core tumor region (yellow), generating the outer region by doubling the core circle's radius, and delimiting the border region according to high smooth muscle actin (SMA) expression. To improve reproducibility with non-round tumor shapes, polygons were extracted from the circu-lar delimitation (red). (**c**) Final binary masks for all three tumor regions.
